# The mitochondrial genome of *Nephrotoma Tenuipes* (Diptera Tipulidae)

**DOI:** 10.1080/23802359.2019.1667271

**Published:** 2019-09-19

**Authors:** Jinlong Ren, Chunmin Zhang, Wencheng Chang, Ding Yang

**Affiliations:** a College of Plant Protection, China Agricultural University, Beijing, China;; b Shanghai Agricultural Technology Extension and Service Centre, Shanghai, China

**Keywords:** Mitochondrial genome, Tipulidae, Phylogenetics, *Nephrotoma*

## Abstract

The crane fly *Nephrotoma tenuipes* belongs to family Tipulidae. The mitogenome of *N. tenuipes* was sequenced, the new representative of the mitogenome of the family Tipulidae. The nearly complete mitogenome is 14,851 bp totally, consisting of 13 protein-coding genes, 2 rRNAs and 22 transfer RNAs. All genes have the similar locations and strands with that of other published species of Tipulidae. The nucleotide composition biases toward A and T, which together made up 76.04％of the entirety. Bayesian inference analysis strongly supported the monophyly of Tipuloidea. It suggested that the phylogenetic relationship within Tipuloidea is Pediciidae + (Limoniidae + (Tipulidae + Cylindrotomidae)).

## Introduction

Superfamily Tipuloidea is one of the highly diversified groups in Diptera with 15,485 recognized species in 703 genera and subgenera (Yeates and Wiegmann 2005; De Jong et al. 2008; Oosterbroek 2019). This superfamily divided into four families, Cylindrotomidae, Limoniidae, Tipulidae, and Pediciidae. Among them, the Tipulidae is the second largest family, including 3792 taxa. The mitochondrial (mt) genomes have been used widely for phylogenetic studies of insects (Cameron 2014). To date, there are 9 complete or nearly complete Tipuloidea mitochondrial genome sequences in GenBank, Among them, three species belong to Tipulidae and only one genus *Tipula*. However, Tipulidae has 124 genera/subgenera. Hence, the more tipulid flies need to be sequenced in order to clarify the phylogeny of Tipuloidea. This paper adds new sequencing data for the family Tipulidae.

The specimens of *Nephrotoma tenuipes* (Riedel, 1910) (Diptera: Tipulidae) (Collecting information: Burqin, Xinjiang, China, 48.56°N, 87.44°E, 1208.9 m, 2016.VII.23, Jinlong Ren.) used for this study were stored in the Entomological Museum of China Agricultural University (CAU, specimen accession number: CAURJL20190302). The total genomic DNA was extracted from the whole body (except for head, wings, and hypopygium) of the specimen using the QIAamp DNA Blood Mini Kit (Qiagen, Germany) and stored at -20 °C until needed. The mitogenome was amplified and sequenced as described in our previous study (Wang et al. 2016). Genbank accession number of sequenced specimen: *Tipula abdominalis* (N861743.1), *Tipula cockerelliana* (NC_030520), *Nephrotoma tenuipes* (MN053900), *Tipula melanomer- a gracilispina* (MK864102), *Cylindrotoma* sp. (KT970060.1), *Symplecta hybrida* (NC_030519), *Paradelphomyia* sp. (KT970061.1), *Limonia phragmitidis* (MK673118), *Rhipidia chenwenyoungi* (KT970063.1), *Pedicia* sp. (KT970062.1), *Trichocera bimacula* (JN861750.1), *Paracladura trichoptera* (NC_016173) and *Sylvicola fenestralis* (NC_016176).

The nearly complete mitogenome of *N. tenuipes* is 14,851 bp. It encoded 13 PCGs, 22 tRNA genes and 2 rRNA genes and the control region could not be sequenced in this study and were similar with related reports before (Wang et al. 2016; Zhang et al. 2016; Ren et al. 2019). All genes have similar locations and strands with that of other published Tipuloidea species. The nucleotide composition of the mitogenome was biased toward A and T, with 76.04% of A + T content (A = 38.32%, T = 37.72%, G = 9.53%, C = 14.43%). The A + T content of PCGs, tRNAs, and rRNAs is 75.03, 76.48, and 79.32%, respectively. The total length of all 13 PCGs of *N. tenuipes* is 11,252 bp. Four PCGs (*NAD2*, *NAD3, ATP8,* and *NAD6*) initiated with ATT codons, and seven PCGs (*ATP6*, *COX2*, *COX3*, *NAD4, NAD4L*, *NAD5,* and *CYTB*) initiated with ATG codons, *COX1* and *NAD1* initiated with TCG and ATA as a start codon, respectively.

**Figure 1.**
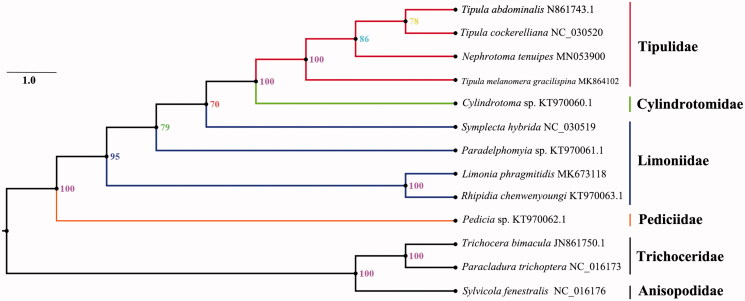
Bayesian phylogenetic tree of 13 Diptera species. The posterior probabilities are tagged at each node. Bayesian analyses were conducted using MrBayes on XSEDE 3.2.6 (CIPRES). The best-fit partitioning scheme and substitution models for partition were determined using PartitionFinder2 on XSEDE (CIPRES). The MrBayes analysis was performed using GTR + I + G models and run 10 million generations for dataset (13-taxon sampling, PCG123 + RNA).

Phylogenetic analysis was performed based on the nucleotide sequences of 13 PCGs and rRNAs from 13 Diptera species. Bayesian (BI) analysis generated the phylogenetic tree topologies based on the PCGs and rRNAs matrices (Figure 1). According to the phylogenetic result, it showed that the monophyletic Tipuloidea was assigned to be the sister group to the clade of Trichoceridae and Anisopodidae. Tipulidae is the sister group of Cylindrotomidae and Pediciidae is a basal clade of Tipuloidea. The *Nephrotoma* has closed relationship with the subgenera *Tipula* (*Nippotipula*) and *Tipula* (*Acutipula*). The phylogenetic relationship within Tipuloidea is very clear with Pediciidae + (Limoniidae + (Tipulidae + Cylindrotomidae)). This relationship was supported by previous studies (Petersen et al. 2010; Zhang et al. 2016).
